# Evaluation of Three Viral Capsid Integrity qPCR Methods for Wastewater-Based Viral Surveillance

**DOI:** 10.1007/s12560-024-09627-x

**Published:** 2025-01-06

**Authors:** Jessica L. Kevill, Kata Farkas, Kate Herridge, Shelagh K. Malham, Davey L. Jones

**Affiliations:** 1 School of Environmental and Natural Sciences, Bangor University, Bangor, Gwynedd LL57 2UW UK; 2 School of Ocean Sciences, Bangor University, Menai Bridge, Anglesey, LL59 5AB UK

**Keywords:** Capsid integrity, Environmental surveillance, Pathogen viability, Public health risk, Viral persistence, Wastewater-based epidemiology

## Abstract

Capsid Integrity qPCR (CI-qPCR) assays offer a promising alternative to cell culture-based infectivity assays for assessing pathogenic human virus viability in wastewater. This study compared three CI-qPCR methods: two novel (Crosslinker, TruTiter) and one established (PMAxx dye). These methods were evaluated on heat-inactivated and non-heat-inactivated ‘live’ viruses spiked into phosphate-buffered saline **(**PBS) and wastewater, as well as on viruses naturally present in wastewater samples. The viral panel included Human adenovirus 5 (HAdV), enterovirus A71 (EV), hepatitis-A virus (HAV), influenza-A H3N2 (IAV), respiratory syncytial virus A2 (RSV), norovirus GI, norovirus GII, and SARS-CoV-2. All three methods successfully differentiated between degraded, heat-inactivated, and live viruses in PBS. While all three methods were comparable for HAdV and norovirus GI, PMAxx detected significantly lower gene copies for EV and IAV. In spiked wastewater, PMAxx yielded significantly lower gene copies for all heat-inactivated viruses (HAdV, EV, HAV, IAV, and RSV) compared to the Crosslinker and TruTiter methods. For viruses naturally present in wastewater (un-spiked), no significant difference was observed between PMAxx and TruTiter methods. Intact, potentially infectious viruses were detected using both PMAxx and TruTiter on untreated and treated wastewater samples. A comparative analysis of qPCR data and TEM images revealed that viral flocculation of IAV may interfere with capsid integrity assays using intercalating dyes. In summary, our findings not only advance the development of more effective methods for assessing viral viability in wastewater, but also highlight the potential of CI-qPCR techniques to enhance early warning systems for emerging pathogens, thereby strengthening public health preparedness and response strategies.

**Supplementary Information:**

The online version contains supplementary material available at 10.1007/s12560-024-09627-x.

## Introduction

Wastewater-based epidemiology (WBE) allows for the quantitative detection of disease-causing pathogens excreted in urine and faeces, providing valuable insights into community-level health (O’Keeffe, 2021; Sims & Kasprzyk-Hordern, 2020). The adoption of WBE has significantly increased since the SARS-CoV-2 pandemic (Barcellos et al., 2023), when it emerged as a cost-effective, non-invasive monitoring tool deployed globally (Agrawal et al., 2022; Ahmed et al., 2020; Brunner et al., 2022; Dzinamarira et al., 2022; Hillary et al., 2021; Kumar et al., 2020; Yu et al., 2022). The process of wastewater monitoring involves collecting samples, concentrating target pathogens, extracting genomic material, and employing molecular methods such as next generation sequencing and quantitative polymerase chain reaction (qPCR) to detect pathogens and establish disease prevalence. The SARS-CoV-2 pandemic highlighted the value of WBE, enabling public health authorities to gain insights into disease trends (Brunner et al., 2022), and make more informed decisions, complementing data generated by traditional clinical surveillance (Hrudey & Conant, 2022). Through wastewater surveillance, researchers can identify the emergence of new variants, track virus transmission, and estimate the true prevalence of infection within a community (Bowes et al., 2023; Hrudey & Conant, 2022; O’keeffe, 2021; Prado et al., 2021; Sims & Kasprzyk-Hordern, 2020). This information can then be used to inform and guide targeted interventions, allocate resources more efficiently, and enhance overall pandemic preparedness and response to disease outbreaks.

Beyond the SARS-CoV-2 pandemic, WBE offers insights into other human health concerns, including anti-microbial-resistant bacteria and fungi (Assress et al., 2021; Cuetero-Martínez et al., 2022; Jari et al., 2022; Kataki et al., 2022; Leonard et al., 2018a, 2018b; Zhang et al., 2021), in addition to human viruses (Beyer et al., 2020; Fantilli et al., 2023; Farkas et al., 2018a, 2018b; Farkas et al., 2018a, 2018b; Huang et al., 2022; Kitakawa et al., 2023; Rachida & Taylor, 2020; Tedcastle et al., 2022; Toribio-Avedillo et al., 2023). Pathogens commonly found in wastewater pose a significant public health risk, as sewage is regularly discharged into the environment, particularly after heavy rainfall events (Perry et al., 2024). Consequently, the public may encounter contaminated water, which can potentially result in a range of infections from skin infections, respiratory illnesses, diarrhoea, and vomiting (Leonard et al., 2018a, 2018b). To better assess the public health risk associated with sewage discharge, it is vital to understand the survival and persistence of pathogens in wastewater, particularly the viability of viruses. While some studies have explored this area (Canh et al., 2022; Fongaro et al., 2013, 2015; Kevill et al., 2024a, 2024b; Leifels et al., 2021; Rimoldi et al., 2020; Stobnicka-Kupiec et al., 2022), it remains largely under researched in the context of WBE. Unlike bacteria, which can be readily cultured from wastewater (Tiwari et al., 2022), assessing viral viability presents unique challenges (Dolskiy et al., 2020).

Viruses cannot replicate outside a living host cell, necessitating the use of cell cultures to assess true viral infectivity. However, isolating viruses from environmental samples for infectivity assays is often complicated by contaminants, requiring carefully balanced antibiotic use to prevent cytotoxic effects (Schmidt et al., 1978). Cell culture requires specialist equipment (Lee et al., 2013), established and well-managed cell lines, in addition to staff expertise. These requirements, coupled with the 4- to 14-day timeframe for generating infectivity data, can hinder rapid public health interventions. While cell culture represents the ‘gold standard’ for assessing viral viability, these limitations highlight the need for alternative methods, especially when dealing with potential pandemic-causing viruses in wastewater.

To address these challenges, capsid integrity qPCR (CI-qPCR) assays (also known as viability assays) have been developed either using intercalating dyes (Da Graça Pedrosa De Macena et al., 2022; Leifels et al., 2019; Prevost et al., 2016; Randazzo et al., 2018a, 2018b; Reyneke et al., 2017), DNAse or RNAse enzymes (Walker et al., 2019), or protein-coated magnetic beads (Farkas et al., 2018a, 2018b). Intercalating dyes function by binding to free genomic material and rendering it unusable for use in downstream applications (e.g. qPCR) (Fittipaldi et al., 2012). Intercalating dye-based methods have been used to assess viral viability in food production (Moreno et al., 2015), drinking water (Fongaro et al., 2013; Iaconelli et al., 2017; Prevost et al., 2016; Randazzo et al., 2018a, 2018b; Razafimahefa et al., 2021), environmental samples (Da Graça Pedrosa De Macena et al., 2022; Leifels et al., 2021; Stobnicka-Kupiec et al., 2022), for evaluating disinfection strategies (Leifels et al., 2015; Shirasaki et al., 2020), and in wastewater (Gyawali & Hewitt, 2018; Kevill et al., 2024a, 2024b; Leifels et al., 2016; Randazzo et al., 2019; Stobnicka-Kupiec et al., 2022).

Despite their potential, capsid integrity assays for wastewater-associated viruses are not yet well established. This gap in methodology is particularly concerning given the increasing frequency of extreme weather events driven by climate change (Rahmani & Mohammad, 2024; Rudd et al., 2023), which is likely to escalate sewage discharge incidents (Perry et al., 2024) and, consequently, human exposure to waterborne pathogens. Therefore, it is essential to establish reliable CI-qPCR assays to inform the risk assessment of sewage-derived viruses, especially in situations where traditional cell infectivity assays are impractical. Previous research has demonstrated the efficacy of PMAxx dyes in distinguishing between degraded viruses and live, intact, potentially infectious viruses in wastewater for enterovirus (EV), norovirus GI and GII, and SARS-CoV-2 (Kevill et al., 2024a, 2024b). However, the PMAxx method's reliance on LED lights for activation may limit its applicability in highly turbid samples or laboratories lacking the necessary equipment. Therefore, it is important to explore alternative capsid integrity assays that do not require light exposure. Currently, Promega™ offers intercalating dye products (Crosslinker and TruTiter) that do not require the use of an LED light to activate them. Unlike the light exposure step required by PMAxx dyes, Crosslinker, and TruTiter intercalating dyes utilise a heat step. Since they do not require specialised equipment, these alternative intercalating dyes could serve as practical substitutes for PMAxx dyes. The aim of this study was, therefore, to compare PMAxx, Crosslinker, and TruTiter in CI-qPCR assays for detecting spiked viruses in phosphate-buffered saline (PBS) and wastewater, as well as viruses naturally present in wastewater samples. The primary objective was to evaluate suitable alternatives to light exposure-dependent intercalating dyes. Our secondary aim was to evaluate the aggregation behaviour of HAdV and IAV, addressing potential limitations of capsid integrity assays in the presence of such phenomena.

## Methods

### Viral Stocks

The viruses used in the spiking experiments were purchased commercially. The following viruses were purchased from ATCC®: Human adenovirus 5 (HAdV) (Cat No: VR-5), enterovirus A71 (EV) (Cat No: VR-1775), influenza-A virus H3N2 (IAV) (Cat No: VR-1881), and respiratory syncytial virus A2 (RSV) (Cat No: VR-1540). Norovirus GI was purchased from the Culture Collection of the UK Health Security Agency. Pseudomonas Phi6 was used as a processing control to assess viral recovery percentages, this was cultured in-house following a protocol described previously (Kevill et al., 2022). All virus stocks were stored at − 80 °C and quantified by RT-qPCR prior to use in experiments.

### Virus Spiking into Phosphate-Buffered Saline (PBS)

Viruses were quantified by RT-qPCR prior to spiking. Viruses were suspended in PBS, pH 7.4. The concentration of viruses spiked into one litre of PBS was as follows: HAdV ~ 10^7^ l^−1^, EV ~ 10^10^ 1^–1^, IAV ~ 10^8^ 1^–1^, and norovirus GI ~ 10^5^ 1^–1^. After adding the viruses to the PBS, the sample was divided into two 500 ml aliquots. One aliquot was treated in a water bath at 70 °C for 30 min, producing a 500 ml sample of heat-inactivated viruses with potentially damaged capsids and reduced infectivity. The other 500 ml aliquot was not heat treated, this is referred to as the 'live' sample, as this sample contains intact, potentially infectious viruses. In triplicate for each method (Crosslinker, TruTiter, PMAxx, and qPCR), 5 ml aliquots of the heat-inactivated and live samples were aliquoted into 15 ml tubes prior to sample processing. All sample processing was completed on the day of experimentation.

### Virus Spiking of Wastewater

A 24-h wastewater influent composite sample was collected on the 3rd July 2023 from the Treborth Wastewater Treatment Plant (WWTP), Bangor, North Wales, UK and transported chilled to the lab, where it was stored at 4 °C until spiking. The concentration of viruses spiked was as follows: HAdV ~ 10^7^ l^−1^, EV ~ 10^9^ l^−1^, HAV ~ 10^8^ l^−1^, IAV ~ 10^8^ l^−1^, and RSV ~ 10^7^ 1^–1^. HAV was utilised as a surrogate for norovirus, as at the time of the wastewater experiment, we had no high titre norovirus stock. RSV was included in the study due to its consistent detection in wastewater matrices. Prior to spiking, each virus stock was split into half. One half (500 µl) was directly spiked into 500 ml of wastewater influent, while the other half was heat inactivated at 70 °C for 30 min in a water bath before spiking into 500 ml of wastewater influent, resulting in live (non-heat-inactivated) and heat-inactivated samples, respectively. Heat inactivation was completed prior to spiking, to inactivate the viruses only and not the microbes in wastewater. The wastewater containing heat-inactivated and live viruses were made into 10 ml aliquots, in triplicate for each method (Crosslinker, TruTiter, PMAxx and qPCR). The spiked wastewater samples were processed immediately after preparation.

### Wastewater Sample Collection and Processing

Comparisons of viruses naturally present in wastewater were made using 24-h composite samples collected from three WWTPs (Wrexham, Kimmel Bay, and Treborth) and three hospitals (Ysbyty Maelor, Ysbyty Glan Clwyd, and Ysbyty Gwynedd) in North Wales, UK. Influent samples were collected from the main drain leaving the hospitals (*n* = 9) and entering the WWTPs (*n* = 8), alongside final effluent from the same WWTPs (*n* = 8). Influent and effluent samples were collected on the same day to enable direct comparisons. All samples were collected between November 2023 and May 2024. Once collected, samples were transported chilled to the lab, stored overnight at 4 °C, and processed the following day. Prior to processing, each wastewater sample was divided into three 10 ml aliquots: two for the pretreatment methods (TruTiter, PMAxx), and one for the ‘no treatment’ (qPCR), resulting in a total 78 samples for processing.

To precipitate larger particulate in the wastewater samples, the samples were clarified by centrifugation at 10,000×*g*, 4 °C, for 10 min. The clarified sample was then transferred to a new 50-ml sterile falcon tube and the pellet discarded. Viruses were concentrated by ultracentrifugation using Merck Amicon™ Ultra-15 Centrifugal Filter Units (15 ml, 10 kDa; Fisher Scientific; Cat No: 10781543). A 5 ml (PBS samples) or 10 ml (wastewater samples) aliquot of each sample was spiked with ~ 10^5^ l^−1^ Phi6 processing control to assess viral recovery, and then transferred to a Amicon™ Ultra-15 Centrifugal Filter Unit and centrifuged at 5000×*g* until the retentate reached a final volume of 200 µl (< 1 h). Retentates for qPCR only were stored at 4 °C, while the remaining retentates were pretreated using Crosslinker, TruTiter, or PMAxx dye. All samples were processed following WHO and national biosafety guidelines (WHO, 2004).

### Crosslinker Pretreatment

Crosslinker (Promega Corp., Madison, WI) pretreatment was conducted following the manufacture’s guidelines. Working in a low-light setting (i.e. lights in the biological safety cabinet turned off, overhead lighting turned off, window blinds closed), Crosslinker solution (1 mM in 100% DMSO) was added to 200 µl of viral concentrate at a final concentration of 10 µM. Samples containing Crosslinker were vortexed to mix and incubated at 37 °C for 30 min. Then, 20 µl of 10 × neutralisation buffer was added to each sample, vortexed to mix, and incubated at room temperature for 15 min. Samples were then subject to nucleic acid extraction.

### TruTiter Pretreatment

TruTiter (Promega Corp.) pretreatment was conducted following the manufacturer’s protocol. Working in a low-light setting, TruTiter™ reagent (1 mM in 100% DMSO) was added to the 200 µl viral concentrates, to a final volume of 10 µM. Samples were vortexed to mix and incubated at 37 °C for 15 min. After incubation, 1 µl of 100 × neutralisation solution B was added to each tube, and vortexed to mix. Tubes were then incubated at room temperature for 5 min, prior to nucleic acid extraction.

### PMAxx Pretreatment

Working in the dark, a 10 mM PMAxx working solution was prepared by diluting PMAxx dye 20 mM with molecular grade H_2_O (Biotium, Cat no: 40069; Biotium Inc., Freemont, CA, USA). PMA enhancer at 1 × concentration was added to each 200 µl viral concentrate and mixed by pipetting. PMAxx dye working solution was added to the concentrates at a final concentration 100 µM, as previously described (Kevill et al., 2024a, 2024b). Samples were incubated in the dark, at 20 °C on a bench top rocker (PMR-30 2D Rocker, Cat No: PMR-30-UK) at 30 oscillations/min. The samples were then exposed to light (465–475 nm) for 20 min at room temperature, using the PMA-Lite™ 2.0 Photolysis Device (Biotium, Cat No: BTE90006). Nucleic acids were extracted directly after light exposure.

### Nucleic Acid Extraction and qPCR

Concentrates with and without capsid integrity treatment had their nucleic acids extracted using the NucliSens extraction reagent kit (BioMerieux, Cat. No. 200293), on a Kingfisher 96 Flex system (Thermo Scientific, Waltham, MA, USA), following a previously described protocol (Kevill et al., 2022). Extracts of 100 µl were stored at − 80 °C until analysis.

All qPCRs (RT-qPCR and CI-RT-qPCR referred to as CI-qPCR) were carried out on a QuantStudio Flex 6 Real-time PCR machine (Applied Biosystems Inc., Waltham, MA, USA). Details of PCR reaction chemistry, primers, probes, RNA and DNA standards, and qPCR conditions were previously published (Kevill et al., 2024a, 2024b; Kevill et al., 2024a, 2024b). All samples were run in duplicates, alongside a standard dilution series ranging from 10 to 10^5^ copies, each plate contained four non-template controls.

### Transmission Electron Microscopy (TEM)

One wastewater 24-h influent composite sample was collected as detailed above. To separate suspended solids from the liquid phase, 400 ml of the composite sample was centrifuged at 10,000×*g*, 4 °C, for 10 min. The clarified sample was then transferred to three sterile containers, resulting in each containing 100 ml. One 100 ml aliquot of clarified wastewater was left un-spiked as a control sample to assess background levels of HAdV and IAV present in the wastewater. The second 100 ml aliquot was spiked with heat-inactivated HAdV and IAV, and a third spiked with live (non-heat-inactivated) HAdV and IAV at concentrations of ~ 10^7^ l^−1^ and ~ 10^6^ l^−1^, respectively. Heat inactivation was carried out as previously detailed. HAdV and IAV were selected for transmission electron microscopy (TEM), as their distinct morphology and size allow them to be easily visualised. HAdV has an icosahedral non-enveloped 90 nm diameter capsid, and IAV has an enveloped capsid of ~ 100 nm in diameter. The un-spiked control, heat-inactivated, and live spiked samples were further split into 50 ml aliquots. One 50 ml of the un-spiked control, heat-inactivated, and live sample was processed following the sample processing method detailed above, this was done in duplicate for each sample; these were the unfiltered samples. The other 50 ml of the un-spiked control, heat-inactivated, and live sample were passed through a sterile 0.22-µM PES syringe filter (Fisherbrand™) prior to sample processing as detailed above. These are the filtered samples. Again, samples were processed in duplicate. After sample processing, the resultant viral concentrates were pretreated with PMAxx dyes (as above), and one duplicate sent to UK Health Security Agency by overnight courier for TEM, and the other used for qPCR analysis. This process resulted in a total of 12 samples: 3 conditions (un-spiked, heat-inactivated, and live) × 2 filtration states (filtered and unfiltered) × 2 analysis methods (qPCR and TEM). Samples were negatively stained on glow-discharged, carbon/pioloform TEM grids using 1.5% (w/v) phosphotungstic acid (Taab Laboratories Equipment Ltd). Digital images were acquired on a JEM1400 TEM (JEOL UK Ltd) fitted with an AMT Nanosprint 12 camera (Deben UK Ltd). This experimental design allowed for the comparison of viral behaviour in filtered and unfiltered wastewater samples under different conditions (heat-inactivated, live, and background levels).

#### Data Analysis

The data generated by qPCR were quality checked following MIQE guidelines (Bustin et al., 2009). The limit of quantification (LOQ) and the limit of detection (LOD) for viral targets in wastewater are as published in Farkas et al. (2022). The gene copies per PCR reaction were converted to gene copies (gc) l^−1^ for viruses, and log_10_ transformed for statistical analysis. The distribution of the data was checked by Shapiro–Wilk test, where *p* ≤ 0.05 indicated a non-normal distribution and *p* ≥ 0.05 indicated the data followed a normal distribution. Normally distributed data were analysed using a one-way ANOVA, followed by multi-comparisons using Tukey pairwise and Tukey simultaneous test for differences of means. Log reductions in viral gene copy number were calculated by obtaining the difference between the gene copies of the qPCR (no pretreatment) and CI-qPCR data. Non-parametric data were analysed using a Kruskal–Wallis test. All analysis was conducted using Minitab® software version 21.4.2 (Minitab Inc., State College, PA).

## Results

### Comparison of Capsid Integrity qPCR Methods on Live and Heat-Inactivated Viruses Spiked into PBS

**Fig. 1:**
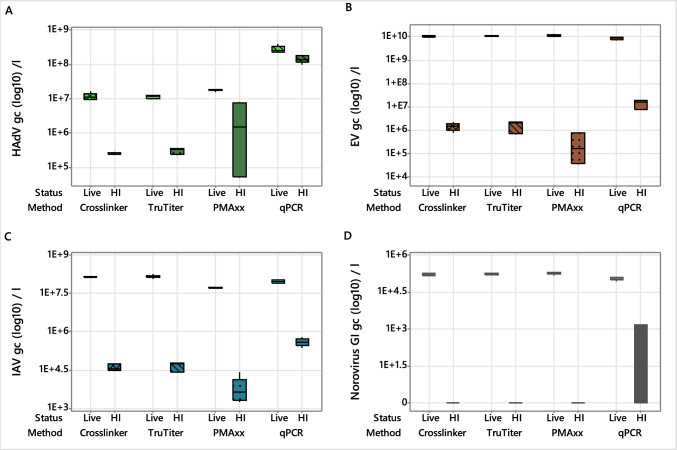
Comparison of viral integrity assays for detecting live (non-heat-inactivated) and heat-inactivated viruses in PBS. Gene copies (gc l^−1^) of HAdV, EV, IAV, and norovirus GI before and after treatment with Crosslinker, TruTiter, or PMAxx assays relative to the untreated control (qPCR)

Statistical analysis revealed significant differences among CI-qPCR methods and standard qPCR in quantifying live and heat-inactivated HAdV, EV, IAV and norovirus GI spiked into PBS (*p* < 0.001; Tables S1, S2). For live (non-heat-inactivated) viruses, the three pretreatment methods (Crosslinker, TruTiter, and PMAxx) yielded similar gene copy numbers for HAdV, EV, IAV, and norovirus GI, (*p* > 0.05; Fig. 1, Table S2). When comparing these pretreatment methods to standard qPCR (no pretreatment) for live EV, IAV, and norovirus GI, no significant differences were found (*p* > 0.05). However, for live HAdV, all pretreatment methods resulted in at least a one-log reduction in gene copies compared to standard qPCR, suggesting partial degradation or incomplete assembly of viruses in this stock.

Heat-inactivated viruses showed markedly different results. For all four viruses (HAdV, EV, IAV, norovirus GI), heat-inactivated samples had significantly lower gene copies (*p* < 0.001) compared to live samples across all pretreatment methods (Crosslinker, TruTiter, and PMAxx; Fig. 1, Table S2). A similar result was also observed when comparing heat-inactivated HAdV, EV, IAV, and norovirus GI pretreatment with Crosslinker, TruTiter, and PMAxx to the heat-inactivated qPCR data (no pretreatment), as pretreated samples had significantly lower gene copies compared to samples that were not pretreated (qPCR) (*p* < 0.002; Fig. 1, Table S2). Comparisons of pretreatment methods show that the three methods gave comparable results for HAdV and norovirus GI spiked into PBS (Fig. 1, Table S2). Comparisons of heat-inactivated EV and IAV showed that the PMAxx method resulted in a significantly lower detection of gene copies than the Crosslinker and TruTiter methods (*p* < 0.001; Fig. 1, Table S2).

### Comparison of Capsid Integrity qPCR Methods on Live and Heat-Inactivated Viruses Spiked into Wastewater

**Fig. 2:**
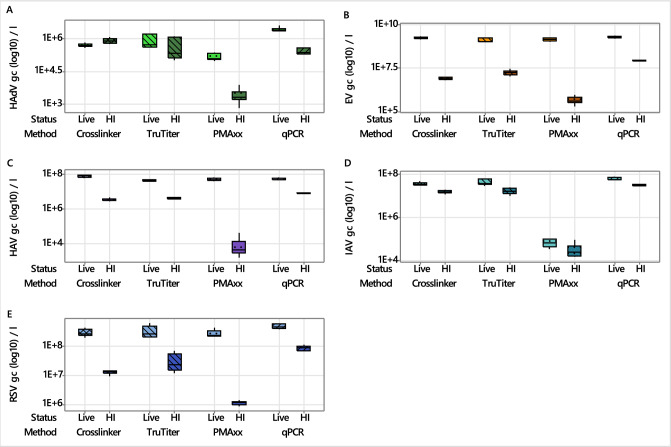
Quantification (gc l^−1^) of heat-inactivated and live (non-heat-inactivated) viruses (HAdV, EV, HAV, IAV and RSV) in spiked wastewater either before (control, qPCR) or after the application of three different capsid integrity pretreatment methods (Crosslinker, TruTiter and PMAxx)

Statistical analysis revealed significant differences among capsid integrity qPCR (CI-qPCR) methods and conventional qPCR in quantifying live (non-heat-inactivated) and heat-inactivated HAdV, EV, HAV, IAV, and RSV spiked into wastewater (Fig. 2, Table S3, *p* < 0.0001). We compared live viruses to their heat-inactivated counterparts for each pretreatment method and those receiving no pretreatment (control, qPCR). All pretreatment methods detected significantly lower gene copies for heat-inactivated viruses compared to qPCR data (*p* < 0.048), except for HAdV using the Crosslinker and TruTiter methods (Fig. 2, Table S4). The Crosslinker pretreatment resulted in significantly lower gene copy detection for all viruses (*p* < 0.05) except for HAdV (Fig. 2, Table S4). Pretreatment with PMAxx resulted in significantly lower gene copies being detected in comparison to the untreated controls for all heat-inactivated viruses (HAdV, EV, HAV, IAV and RSV) (*p* < 0.001; Fig. 2, Table S4). Cross-method comparisons demonstrated that PMAxx pretreatment resulted in significantly lower gene copy detection for all heat-inactivated viruses compared to the Crosslinker and TruTiter methods (*p* < 0.0001; Fig. 2., Table S4). The Crosslinker and TruTiter methods produced comparable results for HAdV, HAV, and IAV (Fig. 2). However, Crosslinker pretreatment resulted in significantly lower gene copy detection for EV (*p* < 0.0001) and RSV (*p* < 0.001) compared to the TruTiter method (Fig. 2, Table S4).

### Comparison of Capsid Integrity qPCR Methods on Viruses Naturally Present in Wastewater

**Fig. 3:**
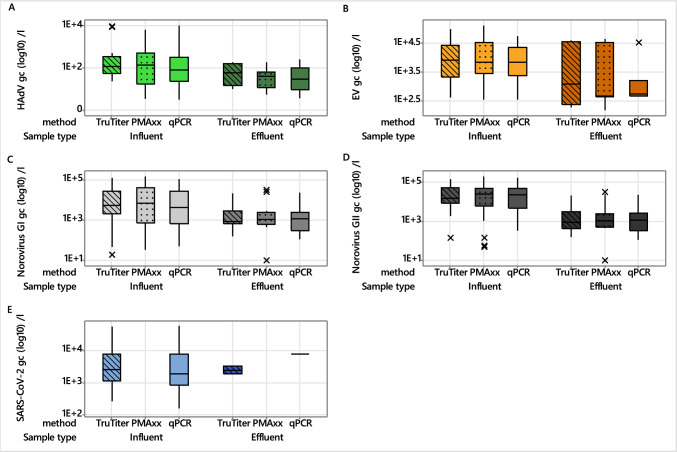
Viral gene copies (gc l^−1^) in wastewater influent and effluent measured after pretreatment with either the TruTiter or PMAxx viral integrity methods. The pretreatment methods were used to assess the integrity of HAdV, EV, norovirus GI, norovirus GII, and SARS-CoV-2 in comparison to samples receiving no pretreatment (qPCR, control). Outliers in the data are indicated with an ×

Wastewater samples were screened in duplicate for the presence of HAdV, EV, IAV, norovirus GI and GII, SARS-CoV-2, and RSV using the TruTiter and PMAxx pretreatment methods and by conventional qPCR (i.e. no pretreatment control). All samples were negative for IAV and RSV. The mean gene copy number of the combined influent and effluent data for HAdV, EV, norovirus GI, and GII showed no significant differences between the two pretreatment methods (Kruskal–Wallis; HAdV *p* = 0.531; EV *p* = 0.459; norovirus GI *p* = 0.985; norovirus GII *p* = 0.997). PMAxx pretreatment resulted in no detection of SARS-CoV-2 in all wastewater samples (Fig. 3). Furthermore, a Kruskal–Wallis test showed no significant difference between the TruTiter and qPCR data for SARS-CoV-2 (*p* = 0.422). HAdV levels showed no significant difference between influent and effluent samples across all methods (TruTiter *p* = 0.121; PMAxx *p* = 0.087; qPCR *p* = 0.091), with reductions all being less than 1-log_10_ unit (Fig. 3A). For EV, effluent showed significantly lower detection rates than influent wastewater (Fig. 3B) for the qPCR (*p* = 0.006) and PMAxx (*p* = 0.05) treatments, while the TruTiter data showed a similar trend but was non-significant (*p* = 0.717). Differences in the copy number of norovirus GI in influent and effluent wastewater were significant for all methods (TruTiter *p* = 0.018; PMAxx *p* = 0.045; qPCR *p* = 0.039), with the effluent having lower detectable gc l^−l^ values (Fig. 3C). A similar result was seen for norovirus GII (TruTiter *p* < 0.001; PMAxx *p* < 0.001; qPCR *p* < 0.001; Fig. 3D). SARS-CoV-2 comparisons between influent and effluent were not possible for PMAxx due to non-detection; however, similar gc l^−l^ values were seen in the influent and effluent values for the TruTiter (*p* = 0.24) and qPCR (*p* = 0.77) methods (Fig. 3E).

### Behaviour of Viruses in Wastewater

**Fig. 4:**
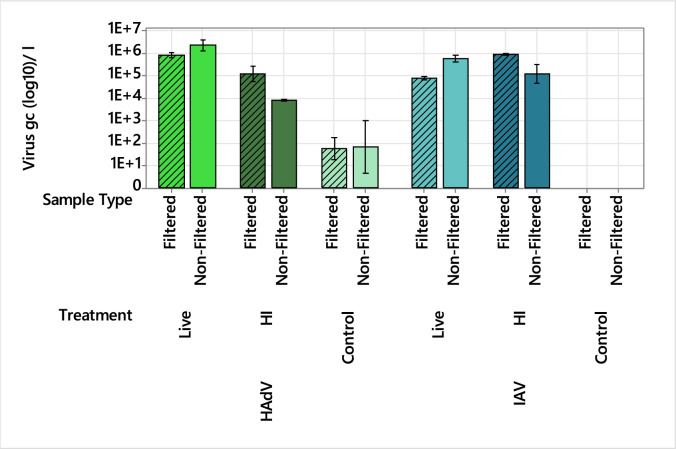
Impact of filtration and heat inactivation on the detection of HAdV and IAV spiked into wastewater. Gene copy concentrations (gc l^−1^) of live and heat-inactivated (HI) HAdV (green) and IAV (blue) in filtered (diagonal line) and non-filtered wastewater, compared to non-spiked controls. All samples were pretreated with PMAxx dyes before nucleic acid extraction. Error bars represent the standard error of the mean

To investigate the behaviour of heat-inactivated and live (non-heat-inactivated) HAdV and IAV in wastewater, their interaction within particulate matter, and how this may influence CI-qPCR assays, we conducted a series of experiments using filtered and unfiltered wastewater. These samples were spiked with live and heat-inactivated viruses (HAdV and IAV), pretreated with PMAxx, and then analysed using qPCR. In parallel, duplicate samples underwent viral imaging using TEM. Gene copy concentrations (gc l^−1^) for live HAdV spiked into wastewater (live) and HAdV naturally present in the wastewater (control) were similar in both the filtered and unfiltered samples (Fig. 4). However, the heat-inactivated (HI) HAdV concentrations were ca. 2-log_10_ lower in the unfiltered samples (Fig. 4). For live IAV, the concentrations detected were ca. 1-log_10_ lower in the filtered sample compared to the unfiltered sample, while the opposite trend was observed in the HI sample. IAV was not detected in the wastewater control (Fig. 4).

**Fig. 5:**
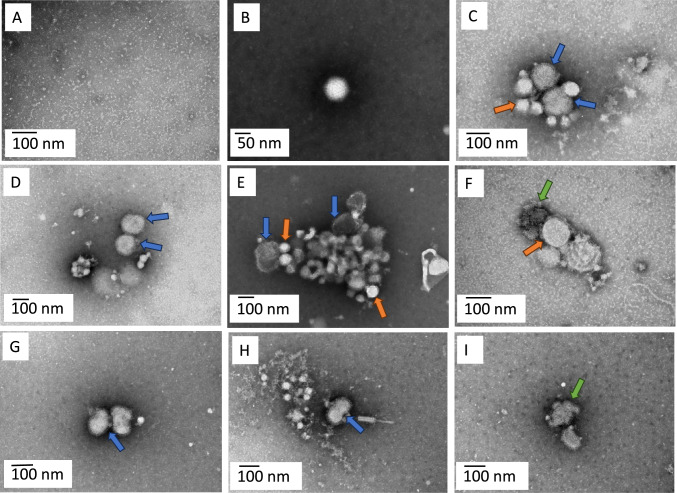
Transmission electron microscopy images of live (non-heat-inactivated) and heat-inactivated HAdV and IAV spiked into wastewater. Panel A—Wastewater control image. Panel B—Live HAdV in non-filtered wastewater. Panel C—Live IAV (blue arrow) floccing and binding to particulate/hydrophobic matter (orange arrow) in filtered wastewater. Panel D—Live IAV (blue arrow) floccing in filtered wastewater. Panel E—Live IAV (Blue arrow) bound to particulate (orange arrow) in filtered wastewater. Panel F—Degraded HAdV (green arrow), bound to particulate (orange arrow) in filtered wastewater. Panel G and H—Heat-inactivated IAV (Blue arrow) floccing. Panel I—heat-inactivated IAV (green arrow)

TEM analysis revealed that HAdV did not appear to flocculate or readily bind to smaller particulate and hydrophobic matter present in the filtered wastewater samples (Fig. 5B). However, we observed aggregation of HAdV capsomeres (Fig. 5F). In contrast, IAV demonstrated a strong tendency to flocculate and bind to particulate matter in both live (Fig. 5C–E) and heat-inactivated filtered samples (Fig. 5G, H).

## Discussion

### Importance of Viral Viability Assessment in Wastewater-Based Epidemiology

WBE studies typically report viral load data obtained by qPCR, enabling researchers to estimate population-level disease prevalence and dynamics (Ciannella et al., 2023; Xagoraraki & O’Brien, 2020). These data can directly inform policy making and risk management, aiding in the prevention of disease outbreaks, as demonstrated during the SARS-CoV-2 pandemic (Corchis-Scott et al., 2021; Prado et al., 2021; Thompson et al., 2020; Wang et al., 2022). However, qPCR data only determine the presence of viral nucleic acids, providing no information about its potential human infectivity.

During the SARS-CoV-2 pandemic, a major research question centred around the potential infectivity of SARS-CoV-2 in wastewater (Foladori et al., 2020; Giacobbo et al., 2021; Kitajima et al., 2020). This assessment proved difficult as wastewater contains a wide range of biological agents and chemicals which may induce non-specific cytotoxicity in culture-based infectivity assays. Consequently, careful sample preparation is needed to remove potential contaminants (e.g. by filtration or the use of antibiotics) (Iaconelli et al., 2017; Schmidt et al., 1978). Furthermore, cell lines used in infectivity assays for emerging pandemic viruses may not be readily available to all laboratories, or wastewater surveillance labs may lack the expertise to manage such cell lines. This is particularly relevant for BSL3 pathogens, such as SARS-CoV-2 and emerging flu strains, such as H5N1. This highlights the need to develop alternative methods to assess viral infectivity, especially in environments where transmission is likely.

Where cell line-based infectivity assays are limited, intercalating dyes can help provide an estimate of potentially viable infectious virions in wastewater samples. As evidenced in this study and elsewhere, encapsulated and potentially infectious human viruses are present in wastewater samples (Da Graça Pedrosa De Macena et al., 2022; Kevill et al., 2024a, 2024b; Leifels et al., 2019, 2021; Randazzo et al., 2019, 2018a, 2018b). Furthermore, methods such as intercalating dyes can provide data for many targets in one assay making them both convenient and cost effective.

Given that many viruses circulate simultaneously, wastewater is expected to contain a wide range of viral targets, making capsid integrity methods ideal for assessing viral viability in WBE studies. While few investigations into human virus viability in WBE exist, the literature indicates a growing popularity of intercalating dyes (EMA, PMA, PMAxx) (Pedrosa De Macena et al., 2022; Kevill et al., 2024a, 2024b; Randazzo et al., 2018a, 2018b; Randazzo et al., 2018a, 2018b). In contrast, the use of ‘gold standard’ cell culture assays are relatively few (Fongaro et al., 2013, 2015; Hewitt et al., 2011; Schlindwein et al., 2010; Wong et al., 2010), and alternative viability methods, such as protein-coated magnetic beads for integrity assays, also remain limited (Farkas et al., 2018a, 2018b; Haramoto et al., 2010).

### Comparison of Capsid Integrity Methods for Viral Viability Assessment

Assessing viral viability in wastewater is crucial for determining associated public health risks, limiting exposure to contaminated water, and preventing potential disease outbreaks (O’Brien & Xagoraraki, 2019; Sims & Kasprzyk-Hordern, 2020). This study investigated two novel capsid integrity methods (Crosslinker and TruTiter) and one established method (PMAxx dye), comparing their effectiveness in detecting a range of sewage-associated viruses of public health importance. Additionally, we applied these methods to evaluate the efficacy of water treatment processes. Our findings indicate that treated final effluent contains potentially viable viruses. This was particularly evident for HAdV, which was detected at similar viral loads in untreated influent and treated effluent, consistent with previous studies (Farkas et al., 2018a, 2018b). EV and norovirus GI and GII exhibited a similar pattern, although water treatment processes did reduce their viral loads. These results align with previous research showing that ca. 48–52% of norovirus GI and GII remains viable after treatment (Cuevas-Ferrando et al., 2022). This data highlights the need for (i) improved water treatment processes to reduce viral loads more effectively, (ii) enhanced risk management strategies to mitigate potential health hazards, and (iii) continued research into viral persistence in wastewater treatment systems. In conclusion, our study emphasises the importance of advanced treatment technologies and robust monitoring protocols to safeguard public health against waterborne viral pathogens.

### Influence of Wastewater Particulates on Viral Detection and Behaviour

Our investigation into heat-inactivated and live HAdV and IAV in filtered wastewater and unfiltered wastewater (i.e. with particulates present) revealed significant differences in viral detection. Live viruses showed higher viral loads in unfiltered samples, while the opposite was seen for the heat-inactivated viruses (i.e. lower viral loads in the unfiltered wastewater). These findings suggest that viral decay and the presence of particulate matter in samples may significantly influence the CI-qPCR assay results.

The TEM imaging indicated that IAV readily flocculates with itself and particulate within the sample, regardless of its state (live or heat-inactivated). This supports previous studies performed in other media (Block et al., 2016; Ksenofontov et al., 2006; Wei et al., 2007). While our study did not reveal clear flocculation evidence for HAdV, previous research has shown that HAdV can form aggregates in the presence of particulates (Wong et al., 2012). Understanding viral flocculation behaviour is important for several reasons including (i) binding to particulates may protect viruses from degradation (Gerba & Betancourt, 2017); (ii) particulate binding may facilitate virus transportation or retention in different environmental matrices; and (iii) particulate-bound viruses could potentially influence disease spread and create new exposure pathways (Wang et al., 2022). None of these potential impacts are fully understood, especially in the case of sewage pollution in waterbodies, necessitating the need for further research in this area.

The flocculation phenomenon also presents challenges for CI-qPCR assays. If degraded viruses are encased in particulates or heavily flocculated, intercalating dyes may not penetrate to the centre of these flocs. This could lead to an overestimation of viable viruses, highlighting a significant limitation of intercalating dye methods. This phenomenon may explain the discrepancies in IAV gene copy detection across pretreatment methods in our spiked wastewater comparisons. Again, it also highlights the need for more research to better understand viral flocculation behaviour across a wide range of sample types, and subsequently how this may inhibit capsid integrity assays.

### Limitations and Future Directions

Our results demonstrate that PMAxx dyes are effective for assessing virus viability across multiple viral targets in wastewater, corroborating findings from other wastewater studies investigating HAdV, Astrovirus, EV, HAV, IAV, norovirus GI, norovirus GII and rotavirus (Pedrosa De Macena et al., 2022; Kevill et al., 2024a, 2024b; Randazzo et al., 2019, 2018a, 2018b). In the PBS samples, the Crosslinker, TruTiter and PMAxx methods performed comparably, successfully differentiating between heat-inactivated, degraded viruses and live, potentially viable, intact viruses. However, in wastewater samples spiked with virus, PMAxx outperformed Crosslinker and TruTiter, detecting significantly lower loads of heat-inactivated viruses. It is worth noting that the concentration of PMAxx was higher in the samples than that of the Crosslinker and TruTiter reagents. The manufacturer suggests that reactions can be optimised to contain between 1 and 50 µM of Crosslinker or TruTiter reagent per reaction, although higher concentrations of reagent may inhibit PCR signal. As a result, for this study, the manufacturer’s guidelines were followed, and 1 µM of Crosslinker and 10 µM of TruTiter were used per reaction. A higher concentration of PMAxx dyes can be used without PCR inhibition and in this study, we used 100 µM per reaction based on previous research in wastewater samples (Kevill et al., 2024a, 2024b).

The similar performance of all methods in PBS samples may be due to (i) the ionic strength of PBS altering the dye binding efficiency to free nucleic acids, or (ii) the absence of chemicals, such as those present in wastewater, which may cause inhibition of intercalating dyes. In un-spiked wastewater samples, where viral loads were lower, TruTiter and PMAxx dyes showed comparable performance. This suggests that viral loads may by a crucial factor when optimising TruTiter assays for wastewater studies. While TruTiter and Crosslinker methods offer a potential alternative to PMAxx, our findings indicate that the concentrations used in this study are likely to provide higher viral viability estimates than PMAxx dyes when viral loads are higher. Further optimisation of these assays is, therefore, necessary for their reliable use in wastewater studies.

## Conclusions

The application of PMAxx dye for detecting potentially viable viruses in wastewater significantly enhances the utility of WBE for public health risk assessments. While our study demonstrates the effectiveness of PMAxx, it also highlights the potential of novel intercalating dyes such as Crosslinker and TruTiter. These new methods, though proven effective in PBS, require further optimisation for wastewater viability assays. Further research is, therefore, needed to better understand the impact of different wastewater sample types on viral behaviour and the limitations of Capsid Integrity qPCR (CI-qPCR) methods in complex matrices. It is important to note that intercalating dyes may lead to an overestimation of viable viruses. Therefore, additional controls and careful experimental design are crucial when employing these methods. Despite these challenges, the integration of CI-qPCR in WBE holds significant promise for expanding our knowledge of infectious risks to wastewater treatment plant workers and the public, in addition to informing and improving risk management strategies when used in conjunction with wastewater-based surveillance programmes.

## Supplementary Information

Below is the link to the electronic supplementary material.

Supplementary file1 (DOCX 52 KB)

## Data Availability

No datasets were generated or analysed during the current study.
