# Supplementary material for: Evaluation of Three Viral Capsid Integrity qPCR Methods for Wastewater-Based Viral Surveillance
Source: Food Environ Virol. 2025 Jan 6;17(1):12. doi: 10.1007/s12560-024-09627-x (PMC11703991; doi:10.1007/s12560-024-09627-x)
Supplement: Supplementary file 1 — Supplementary file1 (DOCX 52 KB) [file 12560_2024_9627_MOESM1_ESM.docx]

**Supplementary Materials**

**Evaluation of three viral capsid integrity qPCR methods for wastewater-based viral surveillance**

Jessica L. Kevill^a,*^, Kata Farkas^a^, Kate Herridge^a^, Shelagh K. Malham^b^, Davey L. Jones^a^

^a^ *School of Environmental and Natural Sciences, Bangor University, Bangor, Gwynedd, LL57 2UW, UK*

^b^ *School of Ocean Sciences, Bangor University, Menai Bridge, Anglesey, LL59 5AB, UK*

*Corresponding author: Jessica L. Kevill ([j.kevill@bangor.ac.uk](mailto:j.kevill@bangor.ac.uk))

**Table S1.** Results of ANOVA analysis performed on the gene copies / l of live and heat-inactivated viruses (Adenovirus (HAdV), Enterovirus (EV), Influenza A Virus (IAV), and Norovirus genotype I) spiked into PBS. Details of model summary are also provided.

|  | **Analysis of Variance** | | | | | | **Model Summary** | | | |
| --- | --- | --- | --- | --- | --- | --- | --- | --- | --- | --- |
| **Virus** | **Source** | **DF** | **Adj SS** | **Adj MS** | **F-Value** | **P-Value** | **S** | **R-sq** | **R-sq(adj)** | **R-sq(pred)** |
| **HAdV** | Method | 7 | 55.328 | 7.904 | 62.0 | <0.001 | 0.3570 | 91.56% | 90.08% | 87.85% |
|  | Error | 40 | 5.099 | 0.1275 |  |  |  |  |  |  |
|  | Total | 47 | 60.427 |  |  |  |  |  |  |  |
| **EV** | Method | 7 | 188.115 | 26.8736 | 431.8 | <0.001 | 0.2494 | 98.69% | 98.47% | 98.12% |
|  | Error | 40 | 2.489 | 0.0622 |  |  |  |  |  |  |
|  | Total | 47 | 190.604 |  |  |  |  |  |  |  |
| **IAV** | Method | 7 | 147.721 | 21.1029 | 625.8 | <0.001 | 0.1836 | 99.10% | 98.94% | 98.70% |
|  | Error | 40 | 1.349 | 0.0337 |  |  |  |  |  |  |
|  | Total | 47 | 149.069 |  |  |  |  |  |  |  |
| **Norovirus GI** | Method | 7 | 282.75 | 40.3924 | 134.5 | <0.001 | 0.5479 | 95.93% | 92.21% | 94.13% |
|  | Error | 40 | 12.01 | 0.3003 |  |  |  |  |  |  |
|  | Total | 47 | 294.76 |  |  |  |  |  |  |  |

**Table S2.** Comparison of capsid integrity qPCR methods of live and heat-inactivated (HI) viruses (Adenovirus (HAdV), Enterovirus (EV), Influenza A Virus (IAV), and Norovirus genotype I) spiked in to PBS. Comparisons were made using Tukey pair-wise tests for differences of means. Significant P values (<0.05) are highlighted in **bold**.

| **Virus** | **Comparison** | **Difference of means** | **95% CI** | **T Value** | **P Value** |
| --- | --- | --- | --- | --- | --- |
| **HAdV** | HI Crosslinker - Live Crosslinker | -1.65 | (-2.309, -0.992) | -8.01 | **<0.0001** |
|  | Live TruTiter - Live Crosslinker | -0.004 | (-0.663, 0.655) | -0.02 | 1 |
|  | Live PMAxx - Live Crosslinker | 0.193 | (-0.466, 0.852) | 0.94 | 0.98 |
|  | Live qPCR - Live Crosslinker | 1.375 | (0.716, 2.034) | 6.67 | **<0.0001** |
|  | HI TruTiter - HI Crosslinker | 0.075 | (-0.584, 0.734) | 0.36 | 1 |
|  | HI PMAxx - HI Crosslinker | 0.518 | (-0.140, 1.177) | 2.51 | 0.22 |
|  | HI qPCR - HI Crosslinker | 2.733 | (2.074, 3.392) | 13.26 | **0.0002** |
|  | HI TruTiter - Live TruTiter | -1.572 | (-2.231, -0.913) | -7.63 | **<0.0001** |
|  | Live PMAxx - Live TruTiter | 0.197 | (-0.462, 0.855) | 0.95 | 0.98 |
|  | Live qPCR - Live TruTiter | 1.379 | (0.720, 2.038) | 6.69 | **<0.0001** |
|  | HI PMAxx - HI TruTiter | 0.444 | (-0.215, 1.103) | 2.15 | 0.4 |
|  | HI qPCR - HI TruTiter | 2.658 | (1.999, 3.317) | 12.9 | **<0.0001** |
|  | HI PMAxx - Live PMAxx | -1.325 | (-1.984, -0.666) | -6.43 | **<0.0001** |
|  | Live qPCR - Live PMAxx | 1.182 | (0.523, 1.841) | 5.73 | **<0.0001** |
|  | HI qPCR - HI PMAxx | 2.215 | (1.556, 2.873) | 10.74 | **<0.0001** |
|  | HI qPCR - Live qPCR | -0.292 | (-0.951, 0.366) | -1.42 | 0.84 |
| **EV** | HI Crosslinker - Live Crosslinker | -3.875 | (-4.335, -3.414) | -26.9 | **<0.0001** |
|  | Live TruTiter - Live Crosslinker | 0.02 | (-0.441, 0.480) | 0.14 | 1 |
|  | Live PMAxx - Live Crosslinker | 0.041 | (-0.419, 0.501) | 0.28 | 1 |
|  | Live qPCR - Live Crosslinker | -0.091 | (-0.551, 0.369) | -0.63 | 0.99 |
|  | HI TruTiter - HI Crosslinker | 0.025 | (-0.435, 0.486) | 0.18 | 1 |
|  | HI PMAxx - HI Crosslinker | -0.914 | (-1.374, -0.453) | -6.34 | **<0.0001** |
|  | HI qPCR - HI Crosslinker | 0.983 | (0.523, 1.443) | 6.83 | **<0.0001** |
|  | HI TruTiter - Live TruTiter | -3.869 | (-4.329, -3.409) | -26.87 | **<0.0001** |
|  | Live PMAxx - Live TruTiter | 0.021 | (-0.439, 0.481) | 0.15 | 1 |
|  | Live qPCR - Live TruTiter | -0.111 | (-0.571, 0.350) | -0.77 | 0.99 |
|  | HI PMAxx - HI TruTiter | -0.939 | (-1.399, -0.479) | -6.52 | **<0.0001** |
|  | HI qPCR - HI TruTiter | 0.958 | (0.497, 1.418) | 6.65 | **<0.0001** |
|  | HI PMAxx - Live PMAxx | -4.829 | (-5.289, -4.369) | -33.53 | **<0.0001** |
|  | Live qPCR - Live PMAxx | -0.132 | (-0.592, 0.329) | -0.91 | 0.98 |
|  | HI qPCR - HI PMAxx | 1.897 | (1.436, 2.357) | 13.17 | **<0.0001** |
|  | HI qPCR - Live qPCR | -2.801 | (-3.261, -2.340) | -19.45 | **<0.0001** |
| **IAV** | HI Crosslinker - Live Crosslinker | -3.556 | (-3.895, -3.217) | -33.55 | **<0.0001** |
|  | Live TruTiter - Live Crosslinker | 0.011 | (-0.328, 0.349) | 0.1 | 1 |
|  | Live PMAxx - Live Crosslinker | -0.419 | (-0.758, -0.080) | -3.95 | **0.007** |
|  | Live qPCR - Live Crosslinker | -0.176 | (-0.515, 0.163) | -1.66 | 0.71 |
|  | HI TruTiter - HI Crosslinker | 0.047 | (-0.291, 0.386) | 0.45 | 1 |
|  | HI PMAxx - HI Crosslinker | -0.884 | (-1.223, -0.545) | -8.34 | **<0.0001** |
|  | HI qPCR - HI Crosslinker | 0.987 | (0.648, 1.325) | 9.31 | **<0.0001** |
|  | HI TruTiter - Live TruTiter | -3.52 | (-3.858, -3.181) | -33.2 | **<0.0001** |
|  | Live PMAxx - Live TruTiter | -0.43 | (-0.769, -0.091) | -4.05 | **0.005** |
|  | Live qPCR - Live TruTiter | -0.187 | (-0.526, 0.152) | -1.76 | 0.65 |
|  | HI PMAxx - HI TruTiter | -0.931 | (-1.270, -0.592) | -8.78 | **<0.0001** |
|  | HI qPCR - HI TruTiter | 0.939 | (0.600, 1.278) | 8.86 | **<0.0001** |
|  | HI PMAxx - Live PMAxx | -4.021 | (-4.360, -3.682) | -37.93 | **<0.0001** |
|  | Live qPCR - Live PMAxx | 0.243 | (-0.096, 0.582) | 2.29 | 0.32 |
|  | HI qPCR - HI PMAxx | 1.87 | (1.532, 2.209) | 17.64 | **<0.0001** |
|  | HI qPCR - Live qPCR | -2.393 | (-2.732, -2.055) | -22.58 | **<0.0001** |
| **Norovirus GI** | HI Crosslinker - Live Crosslinker | -5.214 | (-6.225, -4.203) | -16.48 | **<0.0001** |
|  | Live TruTiter - Live Crosslinker | 0.038 | (-0.973, 1.049) | 0.12 | 1 |
|  | Live PMAxx - Live Crosslinker | 0.048 | (-0.963, 1.059) | 0.15 | 1 |
|  | Live qPCR - Live Crosslinker | -0.167 | (-1.178, 0.844) | -0.53 | 0.99 |
|  | HI TruTiter - HI Crosslinker | 0 | (-1.011, 1.011) | 0 | 1 |
|  | HI PMAxx - HI Crosslinker | 0 | (-1.011, 1.011) | 0 | 1 |
|  | HI qPCR - HI Crosslinker | 1.983 | (0.972, 2.994) | 6.27 | **<0.0001** |
|  | HI TruTiter - Live TruTiter | -5.252 | (-6.263, -4.241) | -16.6 | **<0.0001** |
|  | Live PMAxx - Live TruTiter | 0.01 | (-1.001, 1.021) | 0.03 | 1 |
|  | Live qPCR - Live TruTiter | -0.205 | (-1.216, 0.806) | -0.65 | 0.99 |
|  | HI PMAxx - HI TruTiter | 0 | (-1.011, 1.011) | 0 | 1 |
|  | HI qPCR - HI TruTiter | 1.983 | (0.972, 2.994) | 6.27 | **<0.0001** |
|  | HI PMAxx - Live PMAxx | -5.262 | (-6.273, -4.251) | -16.63 | **<0.0001** |
|  | Live qPCR - Live PMAxx | -0.215 | (-1.226, 0.797) | -0.68 | 0.99 |
|  | HI qPCR - HI PMAxx | 1.983 | (0.972, 2.994) | 6.27 | **<0.0001** |
|  | HI qPCR - Live qPCR | -3.064 | (-4.075, -2.053) | -9.69 | **<0.0001** |
|  |  |  |  |  |  |

**Table S3.** Results of ANOVA analysis and performed on the gene copies / l of live and heat inactivated viruses (Adenovirus (HAdV), Enterovirus (EV), Hepatitis-A, (HAV) Influenza A Virus (IAV), and Respiratory syncytial virus (RSV)) spiked in to wastewater. Details of model summary are also provided.

|  | Analysis of Variance | | | | | | Model Summary | | | |
| --- | --- | --- | --- | --- | --- | --- | --- | --- | --- | --- |
| Virus | Source | DF | Adj SS | Adj MS | F-Value | P-Value | S | R-sq | R-sq(adj) | R-sq(pred) |
| HAdV | Sample Name | 7 | 58.939 | 8.41991 | 138.5 | <0.0001 | 0.246 | 93.09% | 92.42% | 91.47% |
|  | Error | 72 | 4.375 | 0.06788 |  |  |  |  |  |  |
|  | Total | 79 | 63.314 |  |  |  |  |  |  |  |
| EV | Sample Name | 7 | 131.608 | 18.80 | 1463.6 | <0.0001 | 0.113 | 99.30% | 99.23% | 99.14% |
|  | Error | 72 | 0.925 | 0.0128 |  |  |  |  |  |  |
|  | Total | 79 | 132.532 |  |  |  |  |  |  |  |
| HAV | Sample Name | 7 | 126.134 | 18.01 | 587.8 | <0.0001 | 0.175 | 98.28% | 98.11% | 97.88% |
|  | Error | 72 | 2.207 | 0.030 |  |  |  |  |  |  |
|  | Total | 79 | 128.341 |  |  |  |  |  |  |  |
| IAV | Sample Name | 7 | 124.453 | 17.778 | 870.0 | <0.0001 | 0.142 | 98.83% | 98.72% | 98.56% |
|  | Error | 72 | 1.471 | 0.0204 |  |  |  |  |  |  |
|  | Total | 79 | 125.924 |  |  |  |  |  |  |  |
| RSV | Sample Name | 7 | 57.66 | 8.23 | 377.4 | <0.0001 | 0.147 | 97.35% | 97.09% | 96.73% |
|  | Error | 72 | 1.57 | 0.021 |  |  |  |  |  |  |
|  | Total | 79 | 59.232 |  |  |  |  |  |  |  |

**Table S4**. A comparison of capsid integrity qPCR methods for live and heat inactivated (HI) viruses (Adenovirus (HAdV), Enterovirus (EV), Hepatitis-A, (HAV) Influenza A Virus (IAV), and Respiratory syncytial virus (RSV)) spiked in to wastewater. Comparisons were made using Tukey pair-wise tests for differences of means (P< 0.05). The individual confidence level = 99.74% for HAdV, EV, HAV, IAV and RSV.

| **Virus** | **Comparisons** | **Difference of means** | **Slope 95% CI** | **T value** | **P Value** |
| --- | --- | --- | --- | --- | --- |
| HAdV | Live Crosslinker - HI Crosslinker | -0.198 | (-0.543, 0.146) | -1.8 | 0.623 |
|  | Live TrueTiter - HI TrueTiter | 0.346 | (0.002, 0.691) | 3.14 | **0.048** |
|  | Live PMAxx - HI PMAxx | 1.797 | (1.452, 2.141) | 16.3 | **<0.001** |
|  | Live qPCR - HI qPCR | 1.025 | (0.681, 1.370) | 9.3 | **<0.001** |
|  | HI PMAxx - HI Crosslinker | -2.544 | (-2.888, -2.199) | -23.08 | **<0.001** |
|  | HI qPCR - HI Crosslinker | -0.492 | (-0.836, -0.147) | -4.46 | **0.001** |
|  | HI qPCR - HI PMAxx | 2.052 | (1.708, 2.397) | 18.62 | **<0.001** |
|  | HI TrueTiter - HI Crosslinker | -0.365 | (-0.709, -0.020) | -3.31 | **0.03** |
|  | HI TrueTiter - HI PMAxx | 2.179 | (1.835, 2.524) | 19.77 | **<0.001** |
|  | HI TrueTiter - HI qPCR | 0.127 | (-0.217, 0.472) | 1.15 | 0.942 |
|  | Live PMAxx - Live Crosslinker | -0.549 | (-0.893, -0.204) | -4.98 | **<0.001** |
|  | Live qPCR - Live Crosslinker | 0.732 | (0.387, 1.076) | 6.64 | **<0.001** |
|  | Live qPCR - Live PMAxx | 1.28 | (0.936, 1.625) | 11.61 | **<0.001** |
|  | Live TrueTiter - Live Crosslinker | 0.18 | (-0.165, 0.524) | 1.63 | 0.73 |
|  | Live TrueTiter - Live PMAxx | 0.728 | (0.384, 1.073) | 6.61 | **<0.001** |
|  | Live TrueTiter - Live qPCR | -0.552 | (-0.897, -0.207) | -5.01 | **<0.001** |
| EV | Live Crosslinker - HI Crosslinker | 2.3582 | (2.1998, 2.5166) | 46.52 | **<0.001** |
|  | Live TrueTiter - HI TrueTiter | 1.8866 | (1.7282, 2.0450) | 37.22 | **<0.001** |
|  | Live PMAxx - HI PMAxx | 3.5095 | (3.3511, 3.6679) | 69.24 | **<0.001** |
|  | Live qPCR - HI qPCR | 1.3502 | (1.1918, 1.5086) | 26.64 | **<0.001** |
|  | HI PMAxx - HI Crosslinker | -1.2557 | (-1.4141, -1.0973) | -24.77 | **<0.001** |
|  | HI qPCR - HI Crosslinker | 1.0463 | (0.8879, 1.2047) | 20.64 | **<0.001** |
|  | HI qPCR - HI PMAxx | 2.302 | (2.1436, 2.4604) | 45.42 | **<0.001** |
|  | HI TrueTiter - HI Crosslinker | 0.3324 | (0.1740, 0.4908) | 6.56 | **<0.001** |
|  | HI TrueTiter - HI PMAxx | 1.5881 | (1.4297, 1.7465) | 31.33 | **<0.001** |
|  | HI TrueTiter - HI qPCR | -0.7139 | (-0.8723, -0.5555) | -14.08 | **<0.001** |
|  | Live PMAxx - Live Crosslinker | -0.1044 | (-0.2628, 0.0540) | -2.06 | 0.45 |
|  | Live qPCR - Live Crosslinker | 0.0383 | (-0.1201, 0.1967) | 0.76 | 0.995 |
|  | Live qPCR - Live PMAxx | 0.1427 | (-0.0157, 0.3011) | 2.82 | 0.107 |
|  | Live TrueTiter - Live Crosslinker | -0.1392 | (-0.2976, 0.0192) | -2.75 | 0.126 |
|  | Live TrueTiter - Live PMAxx | -0.0348 | (-0.1932, 0.1236) | -0.69 | 0.997 |
|  | Live TrueTiter - Live qPCR | -0.1775 | (-0.3359, -0.0191) | -3.5 | **0.017** |
| HAV | Live Crosslinker - HI Crosslinker | 1.3378 | (1.0931, 1.5825) | 17.09 | **<0.001** |
|  | Live TrueTiter - HI TrueTiter | 1.0128 | (0.7681, 1.2575) | 12.94 | **<0.001** |
|  | Live PMAxx - HI PMAxx | 3.9084 | (3.6637, 4.1531) | 49.92 | **<0.001** |
|  | Live qPCR - HI qPCR | 0.8116 | (0.5668, 1.0563) | 10.36 | **<0.001** |
|  | HI PMAxx - HI Crosslinker | -2.7412 | (-2.9859, -2.4965) | -35.01 | **<0.001** |
|  | HI qPCR - HI Crosslinker | 0.3589 | (0.1142, 0.6036) | 4.58 | **<0.001** |
|  | HI qPCR - HI PMAxx | 3.1001 | (2.8554, 3.3448) | 39.59 | **<0.001** |
|  | HI TrueTiter - HI Crosslinker | 0.0869 | (-0.1578, 0.3316) | 1.11 | 0.953 |
|  | HI TrueTiter - HI PMAxx | 2.8281 | (2.5834, 3.0728) | 36.12 | **<0.001** |
|  | HI TrueTiter - HI qPCR | -0.272 | (-0.5167, -0.0273) | -3.47 | **0.019** |
|  | Live PMAxx - Live Crosslinker | -0.1707 | (-0.4154, 0.0741) | -2.18 | 0.376 |
|  | Live qPCR - Live Crosslinker | -0.1674 | (-0.4121, 0.0773) | -2.14 | 0.401 |
|  | Live qPCR - Live PMAxx | 0.0033 | (-0.2414, 0.2480) | 0.04 | 1 |
|  | Live TrueTiter - Live Crosslinker | -0.2381 | (-0.4828, 0.0066) | -3.04 | 0.062 |
|  | Live TrueTiter - Live PMAxx | -0.0674 | (-0.3121, 0.1773) | -0.86 | 0.989 |
|  | Live TrueTiter - Live qPCR | -0.0707 | (-0.3154, 0.1740) | -0.9 | 0.985 |
| IAV | Live Crosslinker - HI Crosslinker | 0.3808 | (0.1810, 0.5806) | 5.96 | **<0.001** |
|  | Live TrueTiter - HI TrueTiter | 0.4375 | (0.2377, 0.6373) | 6.84 | **<0.001** |
|  | Live PMAxx - HI PMAxx | 0.34 | (0.1402, 0.5398) | 5.32 | **<0.001** |
|  | Live qPCR - HI qPCR | 0.2991 | (0.0993, 0.4989) | 4.68 | **<0.001** |
|  | HI PMAxx - HI Crosslinker | -2.7152 | (-2.9151, -2.5154) | -42.47 | **<0.001** |
|  | HI qPCR - HI Crosslinker | 0.3047 | (0.1049, 0.5045) | 4.77 | **<0.001** |
|  | HI qPCR - HI PMAxx | 3.02 | (2.8201, 3.2198) | 47.24 | **<0.001** |
|  | HI TrueTiter - HI Crosslinker | 0.0198 | (-0.1800, 0.2196) | 0.31 | 1 |
|  | HI TrueTiter - HI PMAxx | 2.7351 | (2.5353, 2.9349) | 42.78 | **<0.001** |
|  | HI TrueTiter - HI qPCR | -0.2849 | (-0.4847, -0.0851) | -4.46 | **0.001** |
|  | Live PMAxx - Live Crosslinker | -2.7561 | (-2.9559, -2.5563) | -43.11 | **<0.001** |
|  | Live qPCR - Live Crosslinker | 0.223 | (0.0232, 0.4229) | 3.49 | **0.018** |
|  | Live qPCR - Live PMAxx | 2.9791 | (2.7793, 3.1789) | 46.6 | **<0.001** |
|  | Live TrueTiter - Live Crosslinker | 0.0765 | (-0.1233, 0.2763) | 1.2 | 0.93 |
|  | Live TrueTiter - Live PMAxx | 2.8325 | (2.6327, 3.0323) | 44.31 | **<0.001** |
|  | Live TrueTiter - Live qPCR | -0.1466 | (-0.3464, 0.0532) | -2.29 | 0.312 |
| RSV | Live Crosslinker - HI Crosslinker | 1.3478 | (1.1414, 1.5543) | 20.4 | **<0.001** |
|  | Live TrueTiter - HI TrueTiter | 1.0655 | (0.8590, 1.2719) | 16.13 | **<0.001** |
|  | Live PMAxx - HI PMAxx | 2.3818 | (2.1753, 2.5883) | 36.05 | **<0.001** |
|  | Live qPCR - HI qPCR | 0.7481 | (0.5417, 0.9546) | 11.32 | **<0.001** |
|  | HI PMAxx - HI Crosslinker | -1.0536 | (-1.2601, -0.8471) | -15.95 | **<0.001** |
|  | HI qPCR - HI Crosslinker | 0.8197 | (0.6132, 1.0261) | 12.41 | **<0.001** |
|  | HI qPCR - HI PMAxx | 1.8733 | (1.6668, 2.0797) | 28.35 | **<0.001** |
|  | HI TrueTiter - HI Crosslinker | 0.3316 | (0.1251, 0.5381) | 5.02 | **<0.001** |
|  | HI TrueTiter - HI PMAxx | 1.3852 | (1.1787, 1.5916) | 20.97 | **<0.001** |
|  | HI TrueTiter - HI qPCR | -0.4881 | (-0.6946, -0.2816) | -7.39 | **<0.001** |
|  | Live PMAxx - Live Crosslinker | -0.0196 | (-0.2261, 0.1869) | -0.3 | 1 |
|  | Live qPCR - Live Crosslinker | 0.22 | (0.0135, 0.4264) | 3.33 | **0.028** |
|  | Live qPCR - Live PMAxx | 0.2396 | (0.0331, 0.4461) | 3.63 | **0.012** |
|  | Live TrueTiter - Live Crosslinker | 0.0492 | (-0.1573, 0.2557) | 0.74 | 0.995 |
|  | Live TrueTiter - Live PMAxx | 0.0688 | (-0.1377, 0.2753) | 1.04 | 0.966 |
|  | Live TrueTiter - Live qPCR | -0.1708 | (-0.3772, 0.0357) | -2.58 | 0.179 |
